# Military Personnel Who Advance Global Surveillance for Infectious Diseases

**DOI:** 10.3201/eid3014.AC3014

**Published:** 2024-11

**Authors:** M. Shayne Gallaway, Jessica Radzio-Basu

**Affiliations:** Defense Health Agency, Armed Forces Health Surveillance Division, Global Emerging Infections Surveillance, Silver Spring, Maryland 20904

**Keywords:** Surveillance, global surveillance, military, infectious diseases, laboratory, serology, epidemiology, art and science, Military Personnel Who Advance Global Surveillance for Infectious Diseases, Carlos J. Anderson, about the cover, United States

**Figure:**
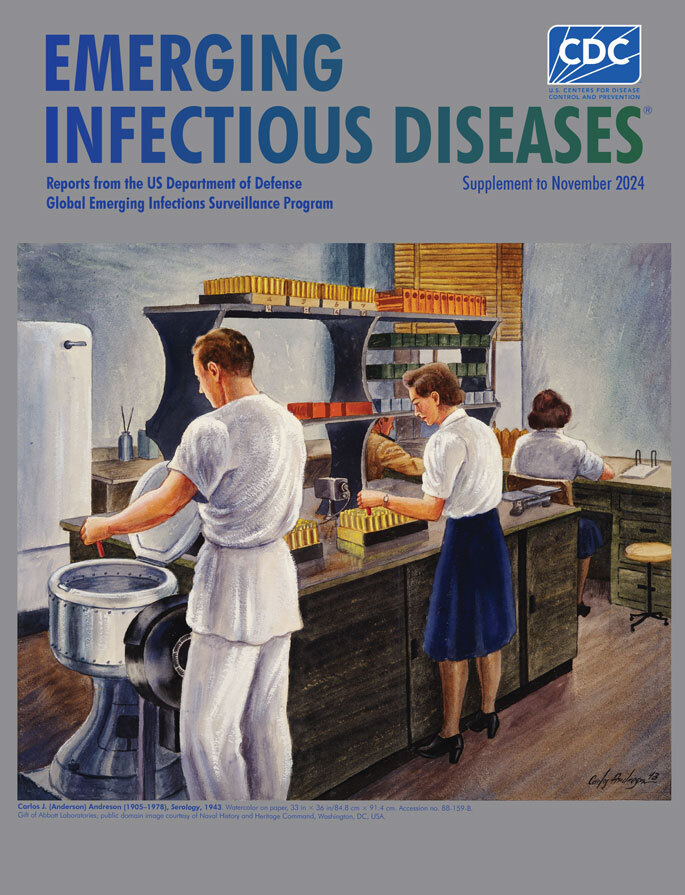
**Carlos J. (Anderson) Andreson (1905–1978). *Serology,* 1943.** Watercolor on paper, 33 in × 36 in/84.8 cm × 91.4 cm. Accession no. 88-159-B. Gift of Abbott Laboratories; public domain image courtesy of Naval History and Heritage Command, Washington, DC, United States.

Soon after World War II began, Abbott Laboratories commissioned Carlos J. Andreson to document medical advancements in US Naval hospitals and showcase the contributions of doctors and scientists to the war effort. Andreson was an American painter, illustrator, graphic artist, and Works Progress Administration artist. He was born in Midvale, Utah, USA, in 1905, as Carlos J. Anderson. Early in his career, he changed the spelling of his last name to Andreson to distinguish himself from other artists with the last name Anderson. His works are currently part of the collections at the Metropolitan Museum of Art, National Museum of American Art, Utah State Fine Arts Collection, and Springville Museum of Art.

Andreson was among the artists commissioned through the New Deal initiatives in the 1930s, which were aimed at providing economic relief during the Great Depression. In particular, the Federal Art Project, under the Works Progress Administration, resulted in the creation of approximately 200,000 works of art across the United States. As part of that effort, Andreson created a series of 24 historical building paintings and drawings. Completed as part of that series was Andreson’s watercolor painting *Serology* (1943), featured on the cover of this supplement.

The work depicts Navy personnel preparing specimens for study in a serology laboratory. In the 1940s, serology was a groundbreaking laboratory technique that played a crucial role in the medical community’s race to find effective treatments amid global upheaval. Serology involved examining blood serum (and other fluids) for the presence of antibodies to specific pathogens. However, at the time, accurate diagnosis using serology was complicated by a lack of standardization, nonspecific reactions, and cross-reacting antibodies. An incomplete understanding of the immune system and the concept of antibody classes made interpretation of results difficult.

During World War II, art played a vital role; it was used for education, public health campaigns, and morale-building. *Serology* highlights the painstaking efforts of laboratory workers and researchers who struggled to make sense of where, when, and how diseases moved through populations, bolstering disease surveillance efforts that would change the practice of public health.

The early 1940s were marked by significant challenges in public health resulting from the global spread of infectious diseases and the beginning of World War II. Considerable challenges and doubts surrounded disease surveillance. During that period and throughout military history, the combination of disease and nonbattle injuries (DNBI) accounted for large numbers of casualties. The proportion of deaths from DNBIs versus battle injuries decreased significantly from the US Civil War (1861**–**1865; 60%) to World War II (25%); however, most Army hospital admissions (95%) during 1941–1945 still resulted from DNBIs. In the 21st Century, DNBIs remain the leading cause of illness and death in conflicts involving the US military. During the 5 major operations making up the Global War on Terrorism (2001–2021), the estimated incidence rate for diseases (e.g., behavioral health, chronic, ill-defined, infectious, respiratory) were almost 3 times higher than the incidence rates for nonbattle injuries and battle injuries.

Since 1946, the US Department of Defense has operated overseas laboratories alongside host-country agencies with the purpose of studying and surveilling infectious diseases of mutual interest during periods of conflict and peace. Those laboratories have made substantial contributions to global health by developing medical countermeasures, assisting with public health emergency responses, and fostering collaborations and friendships within their various regional areas of operation. Serologic tests have proved essential for Department of Defense laboratories to detect infectious diseases such as syphilis, malaria, typhoid fever, and tuberculosis among and within various geographic populations. Globally based US military field hospital laboratories have used serologic testing to manage outbreaks that could infect thousands of troops and render them incapable of serving.

Modern serology-based techniques have evolved from the procedures that Andreson depicted in his painting; the techniques have overcome early limitations associated with diagnosing disease, sample collection, and differentiation of an antibody response (i.e., vaccine vs. natural infection). Modern serology is a key tool used for analyzing human infectious diseases and has applications for public health, disease prevention, clinical diagnosis, and disease management.

Andreson’s painting *Serology* reflects the microscopic interactions between pathogens and the immune system and invites viewers to appreciate laboratory processes and the complexity of nascent science through art. Andreson’s portrayal of a laboratory scene echoes the frustrations of a world still grappling with the rapid spread of disease. The muted palette reflects the somber reality of the limitations in medical technology and public health infrastructure of the time. The interaction of shapes suggests constant observation, symbolizing the early efforts of disease surveillance, which were hampered by limited knowledge and resources. Andreson captures the scientific breakthroughs of the time but also the profound challenges faced by those seeking to control and prevent disease during that period. The artwork’s layered complexity and color tones reflect the concerns generated by incomplete understanding of diseases. The painting serves as a commentary on the scientific advancements of the time and a tribute to the resilience of those striving to improve public health in a time of crisis.
